# Comparative study on main compounds and hypoglycemic effects of dispensing granules of Coptidis Rhizoma and Scutellaria–Coptis herb couple with traditional decoction

**DOI:** 10.1186/s13020-023-00848-z

**Published:** 2023-10-31

**Authors:** Huanhuan Yu, Huilan Tang, Guang Hu, Zhu Chen, Mudan Guo, Bo Jiang, En Zhang, Changhua Hu

**Affiliations:** 1 College of Pharmaceutical Sciences, Southwest University, Chongqing, 400715 China; 2 NMPA Key Laboratory for Quality Monitoring of Narcotic Drugs and Psychotropic Substances, Chongqing Institute for Food and Drug Control, Chongqing, 401121 China; 3 School of Pharmacy and Bioengineering, Chongqing University of Technology, Chongqing, 400054 China

**Keywords:** Dispensing granules, Traditional decoction, Coptidis Rhizoma, Scutellaria–coptis herb couple, Chemical composition, Diabetes

## Abstract

**Background:**

The clinical applications of dispensing granules (DG) have increased dramatically. However, it is controversial whether the DG has the same quality and efficacy compared with traditional decoction (TD). In this study, the contents of main compounds, hypoglycemic effects, and potential mechanism of Coptidis Rhizoma (CR) and Scutellaria–coptis (SC), constituted of a 1:1 mixture of CR and Scutellariae Radix (SR), in the forms of TD and DG were compared.

**Methods:**

The quantitative analysis was performed on an UPLC-PDA method. The 6-weeks-old male *db/db* mice were used as Type 2 Diabetes Mellitus (T2DM) mouse modle to investigate the antidiabetic effects of CR and SC in TD form (CR TD and SC TD), as well as CR and SC in DG form (CR DG and SC DG).

**Results:**

The total content of five alkaloids in CR TD ranged from 71.00 to 78.62 mg, whereas in CR DG it ranged from 38.77 to 53.68 mg in CR DG per 1 g of decoction pieces. Compared to CR TD, CR DG exhibited a 36% reduction on average. For SC samples, the precipitation occurred in the processing of TD but not in the DG, and the relative ratio of alkaloids to flavonoids was determined to be 1:1 in TD and 1:2 in DG. Furthermore, the animal experiments showed that the CR DG (equivalent to 3 g decoction pieces/kg) had almost the same hypoglycemic effect as CR TD when they were administered for 6 weeks. Compared with SC DG (equivalent to 6 g decoction pieces/kg), SC TD showed a better trend in ameliorating T2DM via ameliorating pancreatic structure and function, and activating Akt/AMPK/GLUT4 signaling pathways.

**Conclusion:**

This study indicated that the contents of main compounds were generally higher in CR TD than CR DG originated from the same raw materials. Additionally, changes in the contents of the primary components validated that the compound interactions are exclusive to SC TD during co-decoction, rather than SC DG. The disparate prossing of SC DG and SC TD caused differences both in chemical composition and hypoglycemic effect, suggesting that the substitutability of DG and TD requires further research.

**Supplementary Information:**

The online version contains supplementary material available at 10.1186/s13020-023-00848-z.

## Background

The dispensing granules (DG), as a revolutionary product of traditional Chinese medicines (TCMs), is made from the water extracts of traditional Chinese herbs and further subjected to filtration, concentration, drying, granulation, and packaging [1]. DG is produced by pharmaceutical companies using the decoction pieces of individual traditional Chinese herb, and patients can quickly prepare a decoction containing various granules by mixing and dissolving them with hot water. Traditional decoction (TD) is usually made by boiling several herbs synchronously. Due to the convenience of the innovative product in clinical practice, DG has received increasing attention and adoption from patients and practitioners globally. However, the application of DG has accompanied an inevitable controversy about whether DG can be used as an equivalent substitute for TD. Therefore, it is critical to study the consistency of chemical composition and efficacy of TD and DG.

Coptidis Rhizoma (CR), the dried rhizome of *Coptis chinensis* Franch, *Coptis deltoidea* C.Y. Cheng et Hsiao, and *Coptis teeta* Wall. has been used to treat diarrhea and diabetes for over 2000 years [2, 3]. According to the traditional Chinese medicine theory, the occurrence and development of diabetes is a long-term process, which undergoes a transition from heat to deficiency. CR is known for its ability to clear heat and dry dampness, and often combined with Scutellariae Radix (SR) in classic formulas such as Huanglian Jiedu Decoction and Gegen Qinlian Decoction. These formulas are commonly used to clear stomach heat and relieve intestinal dampness during the heat stage of diabetes [4]. Based on the data mining of clinical observation literatures on the treatment of Type 2 Diabetes Mellitus (T2DM) with TCMs, it is evident that CR stands as one of the most frequently utilized herbs, while Gegen Qinlian Decoction also ranks prominently in terms of usage frequency [5]. SR, the dried root of *Scutellaria baicalensis* Georgi, has various pharmacological activities including anti-inflammation, anti-oxidation, and anticancer [6]. Scutellaria–coptis (SC) herb pair, as the combination of CR and SR at a ratio of 1:1, is a main herb couple in Huanglian Jiedu Decoction and Gegen Qinlian Decoction. Pharmacological studies have revealed that the alkaloids including berberine, coptisine, epiberberine, jatrorrhizine, palmatine in CR, and flavonoids such as baicalin, wogonoside, baicalein, wogonin, oroxylin A in SR are the major bioactive compounds of this herb pair [6, 7]. Recent studies have confirmed that oral administration of CR could ameliorate hyperglycaemia, dyslipidemia, inflammation, and insulin resistance in T2DM, particularly the combination of CR and SR exhibited a synergistic effect on hypoglycemic activity [8, 9]. Furthermore, the hypoglycemic activities of CR and SC may be attributed to various mechanisms, including the increase of insulin secretion and release, inhibition of pancreatic islets cells injury, enhancement of insulin sensitivity, modulation of metabolism-related protein expression and relevant inflammatory factors, and regulation of oxidative stress [2, 8, 10, 11].

Regarding the long-standing clinical applications and increasing researches of CR and SC in diabetes, hypoglycemic effect is widely considered to be one of the important pharmacological effects of them. Although the clinical applications of DG have increased dramatically in recent decades, there has been no systematic comparisons of the quality markers and hypoglycemic effect in vivo of CR or SC between the forms of DG and TD. In this study, the chemical differences were analyzed by quantification of main compounds by ultra-performance liquid chromatography coupled with a photodiode array detector (UPLC-PDA). To objectively compare TD and DG, each TD sample was prepared using the same batch of decoction pieces as the corresponding DG sample. An optimized UPLC method was established for simultaneous quantification of ten main compounds, including coptisine, epiberberine, jatrorrhizine, berberine, palmatine, baicalin, wogonoside, baicalein, wogonin, and Oroxylin A. Furthermore, the comparative studies on the hypoglycemic effects and potential mechanisms between TD and DG preparations of CR and SC was first conducted.

## Materials and methods

### Materials and reagents

Herein, Coptidis Rhizoma (CR) is derived from the dried rhizome of *C. chinensis*, and Scutellariae Radix (SR) is derived from the dried root of *S. baicalensis*. Six batches of CR decoction pieces (CR 01–06), five batches of SR decoction pieces (SR 01–05), eight batches of CR dispensing granules (CR DG01-08), and seven batches of SR dispensing granules (SR DG01-07) were obtained from pharmaceutical manufacturers in China. Detailed information of samples was supplied in Additional file 1: Table S1. All decoction pieces were authenticated and used to produce DG by pharmaceutical manufacturers following the technical requirements for quality control and standard formulation of TCMs dispensing granules [1]. The voucher specimens were deposited at Chongqing Institute for Food and Drug Control, Chongqing, China. It is worth noting that the CR DG01-06 were respectively originated from CR01-06, while the SR DG01-05 were respectively originated from SR01-05. The instruction of DG products indicated that 1 g of CR DG was equivalent to 4.5 g of crude CR, and 1 g of SR DG was equivalent to 2.2 g of crude SR. Chemical standards of coptisine hydrochloride (94.0%), jatrorrhizine hydrochloride (89.5%), berberine hydrochloride (85.9%), palmatine hydrochloride (85.7%), baicalin (94.2%), wogonoside (98.5%), and baicalein (97.9%) were purchased from National Institutes for Food and Drug Control (Beijing, China). Epiberberine (98.92%) was purchased from Chengdu Manst Biotechnology Co., Ltd (Chengdu, China). Wogonin (99.86%) and Oroxylin A (99.83%) were purchased from Zhuhai Anzhe Biotechnology Co., Ltd (Zhuhai, China).

HPLC-grade methanol and acetonitrile were procured from Thermo Fisher Scientific Inc (Fair Lawn, USA). LC MS-grade formic acid was obtained from Merck (Darmstadt, Germany). Ultrapure water (18.2 MΩ) was filtered through a Milli-Q water purification system (Millipore, USA).

### Preparation of traditional decoction

The crude CR and SR were crushed and made into traditional decoction (TD) according to the Standard for Management of TCM Decocting Room in Medical Institutions. Firstly, 10 g of CR or SR was soaked in 100 mL of water for 30 min and boiled for half an hour. After filtrating with a 200-mesh standard sieve, the residue was mixed with 80 mL of water, and followed by decocting for 20 min. Following filtration, the two filtrates were combined and concentrated under vacuum below 60 °C. Afterwards, the concentrated extract was freeze-dried to obtain TD samples. For in vivo experiment, the 30 g of CR and the 60 g mixture of CR and SR with a weight ratio of 1:1 were used to prepare CR TD and Scutellaria–coptis drug pair (SC) TD based on the procedure mentioned above.

### UPLC analysis

Ultra-Performance Liquid Chromatography (UPLC) was conducted using a Waters Acquity UPLC H-Class(Waters, USA) Empower system equipped with an Acquity UPLC SCH™ C18 (100 mm×2.1 mm, 1.7 μm). The mobile phase was composed of A (0.05% formic acid in water, *v/v*) and B (60% methanol and 40% acetonitrile, *v/v*) with a gradient elution: 0–1 min, 88–88% A; 1–3 min 88−80% A, 3–13 min 80% A, 13–15 min 80−64% A, 15–20 min 64–64% A, 20–21 min 64−55% A, 21–30 min 55−45% A. The flow rate of the mobile phase was 0.35 mL/min, and the column temperature was maintained at 35 ℃. The detection wavelength was 270 nm, and the injection volume was 1 µL.

### Quantitative analysis of main compounds

The 10 mg of ten individual standards were respectively dissolved in 10 mL of 50% methanol to prepare standard stock solutions. Subsequently, an aliquot of the individual stock standard solution was combined to form a mixed standard solution. This mixed solution was then diluted with 50% methanol to obtain a range of suitable concentrations, which were utilized for the construction of calibration curves. The TD and DG samples equivalent to 0.1 g decoction pieces were accurately weighed and dissolved in 100 mL of 50% methanol. Subsequently, samples were sonicated for 20 min at room temperature, and the 50% methanol was added to their initial weight after cooling. All solutions were stored at 4 ℃ and were filtered through a 0.22 μm membrane before UPLC analysis.

### Method validation

Peak area versus concentration of each analyte was used to obtain a linear relationship for calibration. The mixed standards solution with the lowest concentration was further diluted to obtain a range of concentrations. The limit of detection (LOD) and limit of quantification (LOQ) were determined at signal-to-noise ratios (S/N) of approximately 3 and 10, respectively. The precision was evaluated by performing six analyzes of a standard solution containing ten standard compounds, and the relative standard deviation (RSD) of the peak area was calculated for each standard compound. The repeatability test was conducted by analyzing six SC TD sample solutions, with the variations expressed by RSD. The stability of sample solution at 4 °C was evaluated by assessing the contents after storage for 0, 2, 4, 8, and 12 h. The recovery test was carried out to asesse the accuracy of the quantitative method.

### Animals

The six-week-old male C57BL/6J homozygous Lepr-/-(*db/db*) mice and C57BL/6J background heterozygous (*db/m*) mice were procured from Better Biotechnology Co., Ltd. (Nanjing, China). The animals were housed in isolated ventilated cages (6 mice/cage) with a 12-h light/dark cycle, relative humidity (55 ± 5%), and constant temperature (23 ± 1 °C). All animals were fed standard rodent pellet food and drinking water *ad libitum*, and acclimatized for one week to use. The animal studies conducted in this research adhered to the Principles for Biomedical Research Involving Animals, established by the Council for International Organizations of Medical Sciences. Additionally, approval for our animal studies was obtained from the Ethics Review Committee for Experimental Animals of Chongqing Institute for Food and Drug Control, and it successfully passed the ethical review under the reference number CQIFDC2022kyhljt01.

### Animal experiment

A total of 36 male *db/db* mice were randomly divided into 6 groups (6 mice in each group): model group (M), M + CR TD group (CR TD), M + CR DG group (CR DG), M + SC TD group (SC TD), M + SC DG group (SC DG), and M + metformin group (P). The normal control group consisted of 6 *db/m* mice (N). CR TD and CR DG were given orally at doses of 0.89 g/kg/d and 0.67 g/kg/d (equivalent to 3 g decoction pieces/kg), respectively. SC TD was given at a dose of 2.31 g/kg/d (equivalent to 6 g decoction pieces/kg/d), while SC DG was given CR DG + SR DG at a dose of 0.67 + 1.36 g/kg/d (equivalent to 3 + 3 g decoction pieces/kg/d) by orally administration. Doses were determined based on previous experiments and literature reports [9, 10]. Mice in the P group were orally administrated metformin at a dose of 200 mg/kg/d [12]. All drugs were suspended in 0.5% carboxymethyl cellulose sodium salt aqueous solution, and were given to mice in relevant groups for 6 consecutive weeks. Mice in the N and M groups were orally administrated with same volume of 0.5% carboxymethyl cellulose sodium salt aqueous solution. During the experiments, measurements of body weight, fasting blood glucose (FBG), and insulin concentration were conducted at different time points. To further evaluate the insulin resistance of mice, the homeostasis model assessment insulin resistance (HOMA-IR) index was calculated using the following formula:

$$\text{HOMA-IR}=\text{fasting blood glucose (mmol/L)} \times \text{insulin (mIU/L)}/22.5$$

To perform oral glucose tolerance tests (OGTT) and insulin tolerance tests (ITT) in vivo, mice were orally administered with glucose (1.0 g/kg of body weight, China Otsuka Pharmaceutical Co., Ltd.) or intraperitoneally injected insulin (0.5 IU/kg, (Beyotime Biotechnology Co., Ltd. Shanghai, China) after 2 h of fasting, respectively. Corresponding blood glucose concentrations were examined at 0, 15, 30, 60, and 120 min following the initial glucose load. The area under the curve (AUC) was calculated to assess the results.

At the end of the experiment, the mice were sacrificed after 2 h under anesthesia with pentobarbital sodium (50 mg/kg). Blood was collected from the eye, allowed to clot for 1 h, and centrifuged at 1500 *g* for 10 min at 4 °C to separate the serum. Separated serum was stored at − 80 °C for biochemical analysis. Histopathological evaluation of pancreatic tissues was conducted after dissection and immersion in 4% paraformaldehyde. The liver tissues and hindlimb skeletal muscle were dissected and immediately frozen in liquid nitrogen for Western blotting analysis.

### Biochemical assays and ELISA detection

Serum levels of total cholesterol (TC), total triglycerides (TG), low-density lipoprotein cholesterol (LDL-C), high-density lipoprotein cholesterol (HDL-C), catalase (CAT), glutathione peroxidase (GSH-Px), and superoxide dismutase (SOD) were assessed using biochemistry reagent kits (Nanjing Jiancheng Bio-Engineering Institute Co., Ltd. Nanjing, China) and malondialdehyde (MDA) were detected by biochemistry reagent kits (Beyotime Biotechnology Co., Ltd. Shanghai, China). The levels of tumor necrosis factor-α (TNF-α), interleukin-1β (IL-1β), and interleukin-6 (IL-6) in serum were assessed using enzyme-linked immunosorbent assay (ELISA) kits (Jingmei Biotechnology Co., Ltd. Jiangsu, China) and a multifunctional enzyme labeling tester (Epoch, American Berten Instruments Co., Ltd. Vermont, America). Certain experimental procedures were performed strictly according to the manufacturer’s protocol.

### Histopathological and immunohistochemical examination

Pancreatic tissue was fixed for 48 h in a 4% paraformaldehyde solution, followed by dehydration and embedding in paraffin. The paraffin-embedded pancreas sections were subjected to deparaffinization in xylene, hydration in a graded alcohol series, and staining with hematoxylin and eosin (H&E) using established protocols. Additionally, immunohistochemical staining for insulin and terminal deoxynucleotidyl transferase (TdT)-dUTP nick-end labeling (TUNEL) staining were performed. An Olympus BX51 photograph system (Tokyo, Japan) was used to record the observations, and an imaging analysis software, Image-Pro Plus 6.0, was adopted to measure and analyze the area. Using Roche Diagnostics’ TUNEL assay kit (Manheim, Germany), the apoptosis was detected according to manufacturer’s instructions. Apoptotic (positive) islet cells are characterized by their dark brown nuclei after TUNEL staining. The positive cells in the 5 noncontiguous high-power fields of vision was counted using the microscope. For each section, three to five islets were counted and an average value was calculated.

For immunohistochemistry, antigens were retrieved from sections following deparaffinization, hydration, and boiling with 0.01 mol/L sodium citrate buffer (pH 6.0) using a microwave for 20 min. The activity of endogenous peroxidase was inhibited through a 10-min incubation with 3% H_2_O_2_ at room temperature. Following this, the sections were treated with 0.1% goat serum for 15 min at room temperature, and then subjected to an overnight incubation at 4 °C with anti-insulin (1:400). This was succeeded by a 15-minute exposure to a secondary antibody (1% biotinylated goat anti-mouse/rabbit IgG) at 37 °C. Horseradish peroxidase-labeled streptomyces ovalbumin was then treated for 15 min at 37 °C, followed by staining with 3,3′-diaminobenzidine (DAB) at room temperature. Nuclei were identified after counterstaining with hematoxylin for 1 min at room temperature. As a negative control, the insulin primary antibodies were replaced with rabbit nonspecific IgG. Using a DP71 fluorescence microscope (Olympus Corporation, Tokyo, Japan), the stainings were visualized at a 400x magnification. Image analysis was facilitated by the Adobe Photoshop SC4 extended software (Adobe, Inc., CA, USA). For semi-quantitative scrutiny of insulin expression in the pancreatic tissue, Image pro Plus 6.0 software (Media Cybernetics, Inc., MD, USA) was employed. From each section, a random selection of three to five distinct fields was chosen for image capture and subsequent average computation for analysis at a magnification of 400x. Automatic measurements were made for the integrated optical density (IOD) of cells that had a positive stain, as well as the corresponding islet areas, from which the averages were extracted to compute the average optical density (IOD/area), indicating the relative quantity of the target protein. Post insulin staining, the islet count for each slide was recorded and the area of the largest islet on each slide was measured for assessing the mean islet size.

### Western blotting analysis

Specimens of liver and skeletal muscle tissues (30–50 mg) were subjected to homogenization and lysis in a volume of 150–250 µl of PMSF-enriched lysis buffer (Beyotime Biotechnology, Shanghai, China) for 30 min on ice. The protein samples, all equivalent in amount (60 µg), were then subjected to electrophoretic separation on polyacrylamide gels composed of 6–10% (*w/v*) sodium dodecyl sulphate, and subsequently transferred to polyvinylidene difluoride (PVDF) membranes (Millipore, Billerica, MA, USA). The PVDF membranes were then subjected to a blocking process using a solution of Tris-buffered saline with 0.1% (*w/v*) Tween 20 (TBS-T), containing 5% defatted milk for 1 h at room temperature, and subsequently hybridized with the intended primary antibodies at 4 °C overnight. This was followed by a room temperature incubation for 1 h with HRP-conjugated secondary antibodies at a dilution of 1:20,000 (supplied by Bioworld Technology, MN, USA). β-actin served as an internal control to ensure equal protein loading. Bands of interest on the blots, which were immunoreactive, were assessed using an ECL detection kit (supplied by Millipore). Signal acquisition was facilitated by the GelDoc XR + IMAGELAB system (Bio-Rad, Hercules, CA, USA). The intensities of these bands were quantified through the use of Quantity One software (Bio-Rad, Hercules, CA, USA). Any fluctuation in band intensity following normalization to β-actin was presented as fold change relative to the reference N control group.

### Statistical analysis

The results were presented as mean ± standard deviation (mean ± SD). The statistical analysis was conducted using SPSS 19.0 software (IBM, Chicago, IL, USA), and group comparisons were performed by one-way analysis of variance (ANOVA). Fisher’s least significant difference (LSD) post hoc test was employed to detect differences between groups when statistically significant differences were observed, with a significance level set at p < 0.05. Significance levels were denoted as *p < 0.05, **p < 0.01, and ***p < 0.001.

## Results

### Method validation

The linearity, correlation coefficient values, LOD, LOQ, precision, stability, repeatability, and recovery of the quantitative method were all examined (Table 1). The high correlation coefficient values (r ≥ 0.9993) of ten analytes (five alkaloids and five flavonoids) suggestted good correlations between concentrations and the peak areas within the tested ranges. The sensitivity was determined by assessing the values of LOD and LOQ. The precision, stability, and repeatability were expressed as RSD varying from 0.23 to 0.59%, 0.32 to 5.67%, and 1.49 to 3.65%, respectively. The recovery rates of ten analytes ranged from 95.32 to 104.75%, with RSD varying from 0.78 to 4.67%. Above results suggestted that the established method exhibits sufficient sensitivity and accuracy for the quantitative analysis of the ten compounds.

**Table 1:** The results of methodological validation

Compounds	Linearity (ug/mL)	r	LOD (ug/mL)	LOQ (ug/mL)	Precision (RSD%)	Stability (RSD%)	Repeatability (RSD%)	recovery (%)	recovery (RSD%)
Coptisine	0.57–57.25	0.9999	0.12	0.41	0.32	1.78	1.93	104.75	1.98
Epiberberine	2.21–44.22	0.9999	0.15	0.48	0.40	0.92	2.95	100.94	4.67
Jatrorrhizine	0.66–13.16	0.9999	0.10	0.33	0.38	1.80	1.54	101.57	1.95
Berberine	0.86–86.24	0.9999	0.22	0.74	0.24	0.55	3.49	102.07	4.24
Palmatine	2.05–41.14	0.9997	0.24	0.81	0.42	2.01	3.52	103.65	1.75
Baicalin	2.34-234.32	0.9999	0.10	0.33	0.30	0.32	3.65	98.52	4.28
Wogonoside	0.95–95.35	0.9999	0.07	0.23	0.26	0.79	1.49	100.37	1.83
Baicalein	0.30-30.23	0.9993	0.09	0.29	0.59	5.67	3.50	98.29	3.46
Wogonin	0.40–7.94	0.9999	0.06	0.20	0.23	1.24	2.75	96.21	4.32
Oroxylin A	0.29–28.91	0.9999	0.09	0.29	0.45	1.50	2.69	95.32	0.78

### Quantitative analysis

**Fig. 1:**
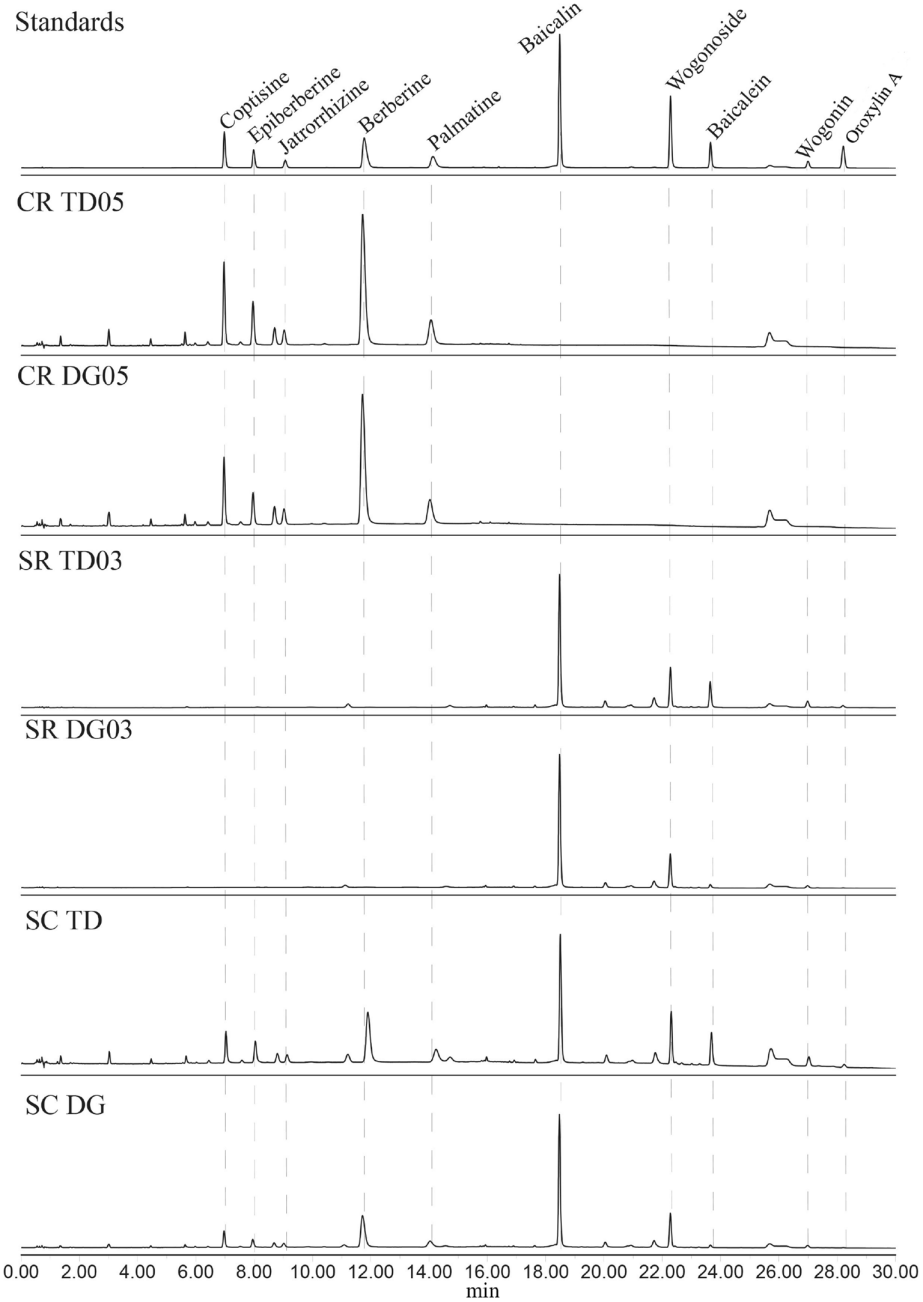
UPLC chromatograms

The major components in Coptidis Rhizoma (CR) and Scutellariae Radix (SR) were well-separated under the optimized chromatographic condition, and the typical UPLC chromatograms of the traditional decoction (TD) and dispensing granules (DG) of CR, SR, and Scutellaria–coptis herb couple (SC) were shown in Fig. 1. According to the extraction rates of TD (Additional file 1: Table S2) and the specifications of DG, the contents of five alkaloids in CR and five flavonoids in SR were expressed as absolute amounts equivalent to 1 g of decoction pieces for parallel comparison (Tables 2 and 3). Compared with each TD sample, the content levels of target compounds were generally lower in corresponding DG samples which was produced by the same batch of decoction piece. The total contents of five alkaloids (berberine, coptisine, epiberberine, jatrorrhizine, palmatine) in CR TD and CR DG respectively ranged from 71.00 to 78.62 mg, and 38.77 to 53.68 mg per 1 g of decoction pieces. The total contents of five flavonoids (baicalin, wogonoside, baicalein, wogonin, oroxylin A) in SR TD and SR DG were from 108.73 to 125.72 mg, and 86.20 to 109.51 mg per 1 g of decoction pieces. The average reduction in total alkaloid content in CR DG is 36% compared to CR TD, whereas the total flavonoid content in SR DG is diminished by 16% relative to SR TD.The distinctions between commercial-scale and laboratory-scale simulations of processing might be one of the reasons for such differences.

**Table 2:** Contents of main alkaloids in CR samples (mg /g decoction piece)

Samples	Coptisine	Epiberberine	Jatrorrhizine	Berberine	Palmatine	Total
TD01	12.90	11.14	2.92	39.35	10.08	76.39
TD02	14.15	12.79	2.86	39.11	9.71	78.62
TD03	12.64	12.08	2.86	38.10	10.19	75.87
TD04	12.74	10.48	2.65	36.21	8.92	71.00
TD05	12.60	11.69	2.75	36.91	9.41	73.36
TD06	13.53	11.84	3.05	39.43	10.49	78.34
DG01	7.13	6.17	2.06	26.48	6.79	48.63
DG02	5.96	5.22	1.58	20.82	5.38	38.96
DG03	5.89	5.17	1.61	20.81	5.29	38.77
DG04	7.81	6.49	2.07	26.26	6.63	49.26
DG05	8.17	6.83	2.15	27.29	7.02	51.46
DG06	8.49	7.05	2.23	28.44	7.26	53.47
DG07	8.47	7.26	2.16	28.56	7.23	53.68
DG08	7.89	6.82	2.13	27.30	7.04	51.18

**Table 3:** Contents of main flavonoids in SR samples (mg/g decoction piece)

Samples	Baicalin	Wogonoside	Baicalein	Wogonin	Oroxylin A	Total
TD01	80.79	19.49	10.17	2.51	1.05	114.01
TD02	94.46	17.29	10.37	2.51	1.10	125.72
TD03	85.32	19.32	10.88	2.76	1.11	119.40
TD04	92.54	18.18	9.62	1.92	0.97	123.22
TD05	80.43	16.78	8.52	2.08	0.92	108.73
DG01	69.42	13.26	2.06	1.09	0.38	86.20
DG02	69.85	13.10	2.52	1.38	0.52	87.37
DG03	83.35	16.16	1.94	1.05	-	102.49
DG04	83.37	16.19	1.87	1.04	-	102.46
DG05	78.26	14.15	3.68	1.85	0.60	98.55
DG06	87.70	16.52	3.17	1.57	0.56	109.51
DG07	84.04	16.39	3.05	1.52	0.51	105.51

-: The content was lower than the LOQ

Based on quantitative results, the CR DG05 and SR DG03 were selected as representative samples of CR and SR for subsequent in vivo experiments. Because their contents of major compounds were close to the mean values of DG samples. Thus, the samples of CR DG05, CR TD05, SC DG consisting of CR DG05 and SR DG03, and SC TD prepared by boiling the decoction pieces of CR 05 and SR 03 together at a ratio of 1:1 were used for comparative study on hypoglycemic effects. The contents of the main compounds in SC DG and SC TD were assessed and were shown in Table 4. The findings of this study indicated a significant reduction in the levels of main components within the SC TD compared to the single herb decoction. The contents of five alkaloids were reduced by 14.5–44.94%, while the five flavonoids were reduced by 19.7–58.6%. However, the above-mentioned changes were not been observed in the DG samples after compatibility, indicating that the interaction between the crude drugs occurred during the boiling process rather than simply mixing and dissolving. Remarkably, it was found that the relative ratio of alkaloids to flavonoids varied from 1:1 in SC TD to 1:2 in SC DG. The change in the relative ratio of active constituents may arise as a result of alterations in the activity.

**Table 4:** The quantitative results of SC samples for in vivo experiment (mg/g decoction pieces)

Samples	Coptisine	Epiberberine	Jatrorrhizine	Berberine	Palmatine	Baicalin	Wogonoside	Baicalein	Wogonin	Oroxylin A	A/F
SC TD	7.82	9.38	2.13	20.32	8.05	35.32	11.36	6.57	1.84	0.89	1:1
SC DG	7.91	6.74	2.11	27.04	7.18	79.69	15.61	1.53	1.03	*	1:2

A/F: The relative ratio of main alkaloids to main flavonoids

*The peak area was lower than the LOQ

### The samples showed different degrees of anti-T2DM effects in diabetic mice

**Fig. 2:**
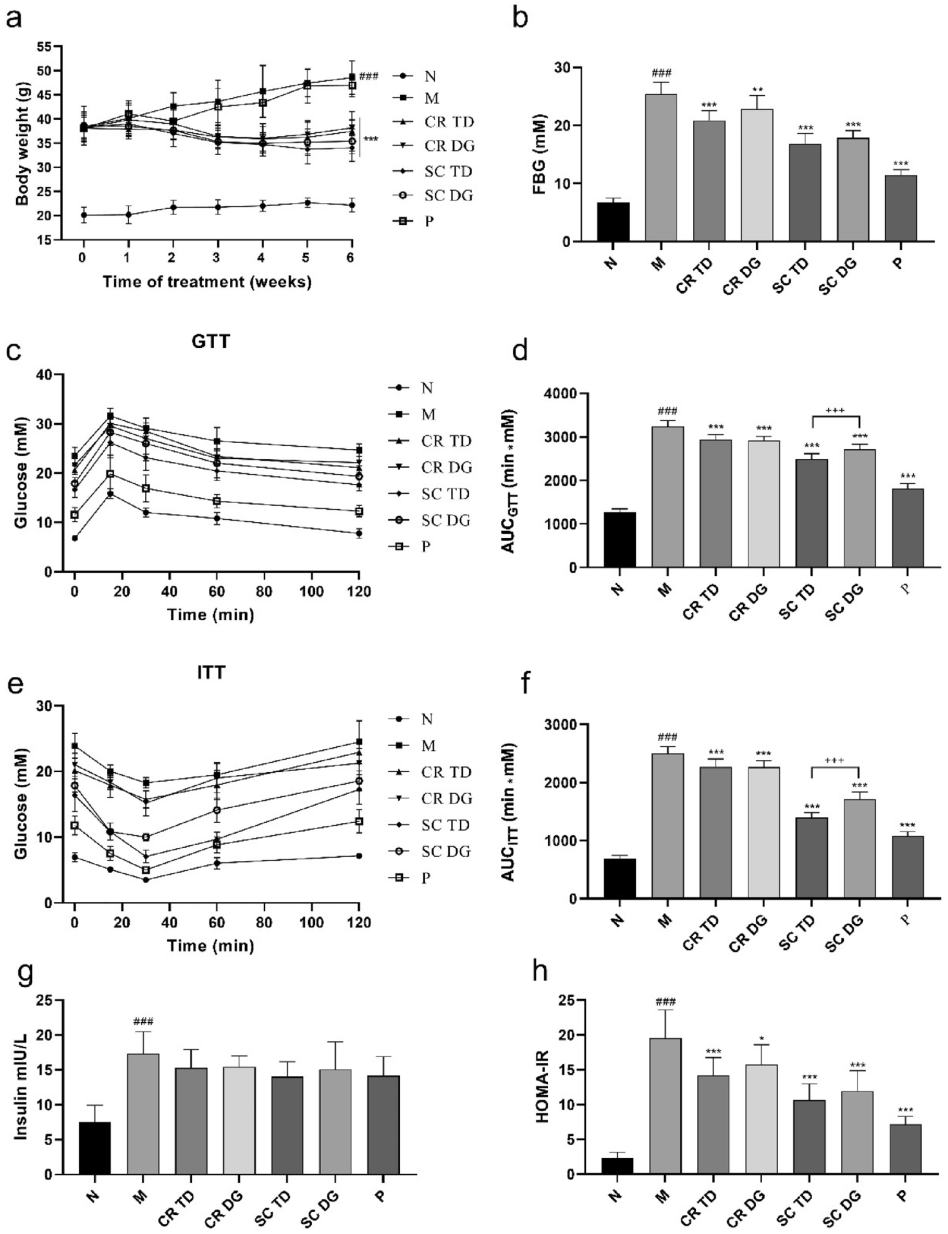
Effects of samples on body weight and glucose metabolism in diabetic mice. **a** Body weight of mice during the treatment. **b** Fasting blood glucose (FBG) of mice in each group at the end of drug treatment. **c** The OGTT was measured on day 36 after drug administration. **d** The AUC of glucose for the OGTT test. **e** The ITT was measured on day 39 after drug administration. **f** The AUC of glucose for the ITT test. **g** The fasting serum insulin level of mice. **h** The homeostasis model assessment-insulin resistance (HOMA-IR) index of mice. N: normal control group (*db/m* mice); M: model group (*db/d*b mice); CR TD, CR DG, SC TD, SC DG, and P: *db/db* mice treated with CR TD, CR DG, SC TD, SC DG, and metformin. Data were expressed as the mean ± SD (*n* = 6 mice per group). ^###^p < 0.001 versus N group; *p < 0.05, **p < 0.01, ***p < 0.001 versus M group; ^+++^p < 0.001 versus SC TD group. Data were analyzed by one-way-ANOVA.

To investigate the hypoglycemic activity in vivo, the *db/db* mouse model of type 2 diabetes was employed. Mice in the model group (M) exhibited notable body weight gain during 6 weeks. Mice treated with metformin (P), a first-line clinical hypoglycemic drug, did not show a significant weight loss effect. However, mice treated with CR TD, CR DG, SC TD, and SC DG showed a significant loss in body weight (p < 0.001) (Fig. 2a). The fasting blood glucose (FBG) detection results showed that all samples and metformin had hypoglycemic effects to different degrees (p < 0.01 or p < 0.001) (Fig. 2b). The present results showed that CR TD, CR DG, SC TD, and SC DG had significant therapeutic effects on hyperglycemia and T2DM-related obesity in *db/db* mice.

The glucose tolerance after drug treatment was evaluated by an oral glucose tolerance test (OGTT) on day 36. The M group showed significantly higher FBG than normal control group (N) during the 120 min OGTT (p < 0.001) (Fig. 2c, d), indicating that the *db/db* mice had impaired glucose tolerance. However, the level of FBG in *db/db* mice was significantly decreased by CR TD, CR DG, and SC DG (p < 0.01 or p < 0.001), especially SC TD and metformin (p < 0.001). The insulin sensitivity after drug treatment was further evaluated using the insulin tolerance test (ITT) on day 39 and found that the insulin tolerance of *db/db* mice was significantly ameliorated in all the tested groups (p < 0.001), among which the effect of SC TD was the most pronounced. It was worth noting that the AUCs of OGTT and ITT in the SC TD group were significantly lower than those in the SC DG group (p < 0.001, Fig. 2e, f). In addition, the mice in the M group exhibited a marked increase in fasting serum insulin (p < 0.001), as well as the homeostasis model assessment-insulin resistance (HOMA-IR) index (p < 0.001) (Fig. 2g, h). After treatment, the HOMA-IR levels were decreased markedly by CR TD, CR DG, and SC DG, especially SC TD (p < 0.01 or p < 0.001) and metformin (p < 0.001), while there was no significant difference in the insulin concentration compared with M group. All these data showed that CR TD, CR DG, SC TD, and SC DG can significantly ameliorate type 2 diabetes in *db/db* mice, and SC TD exerted the strongest effect among them.

### Ameliorative effects of samples on lipid metabolism

**Fig. 3:**
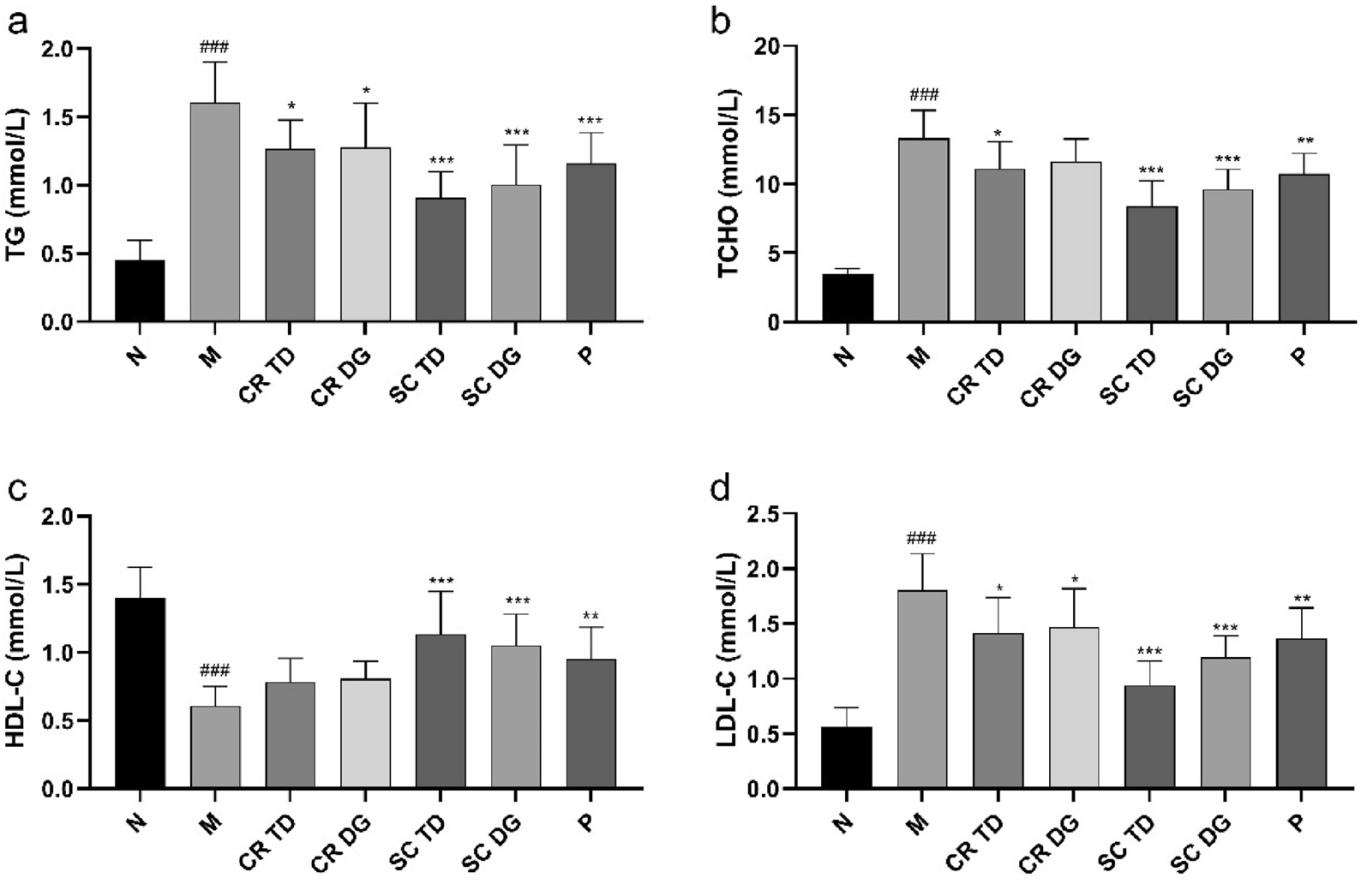
Effects of samples on lipid metabolism in diabetic mice. **a** The levels of triglyceride (TG), **b** total cholesterol (TCHO), **c** high-density lipoprotein cholesterol (HDL-C), and **d** low-density lipoprotein cholesterol (LDL-C) in the serum of diabetic mice. N: normal control group (*db/m* mice); M: model group (*db/db* mice); CR TD, CR DG, SC TD, SC DG, and P: the *db/db* mice treated with CR TD, CR DG, SC TD, SC DG, and metformin. Data were expressed as the mean ± SD (*n* = 6 mice per group). ^###^p < 0.001 versus N group; *p < 0.05, **p < 0.01, ***p < 0.001 versus M group. Data were analyzed by one-way-ANOVA.

Compared with the N group, the levels of triglyceride (TG), total cholesterol (TCHO), and low-density lipoprotein cholesterol (LDL-C) in the M group increased significantly (p < 0.001), while the level of high-density lipoprotein cholesterol (HDL-C) decreased markedly (p < 0.001) (Fig. 3), indicating a severe disorder of lipid metabolism in *db/db* mice. Compared with the M group, the CR TD exhibited moderate influence on TG, TCHO, and HDL-C levels (p < 0.05), while the CR DG only showed ameliorative effects on TG and HDL-C levels (p < 0.05). The SC TD and SC DG showed the most effective amelioration of four indicators in *db/db* mice among all tested groups (p < 0.001). The findings are consistent with prior research, which has demonstrated that both CR and SR contribute to enhancing lipid metabolism in T2DM rats, and the combined extracts exhibited a more pronounced effect than individual herbs, owing to their synergistic properties [8].

### The samples ameliorated the destructed islets in diabetic mice by inhibiting pancreatic decompensation and apoptosis

**Fig. 4:**
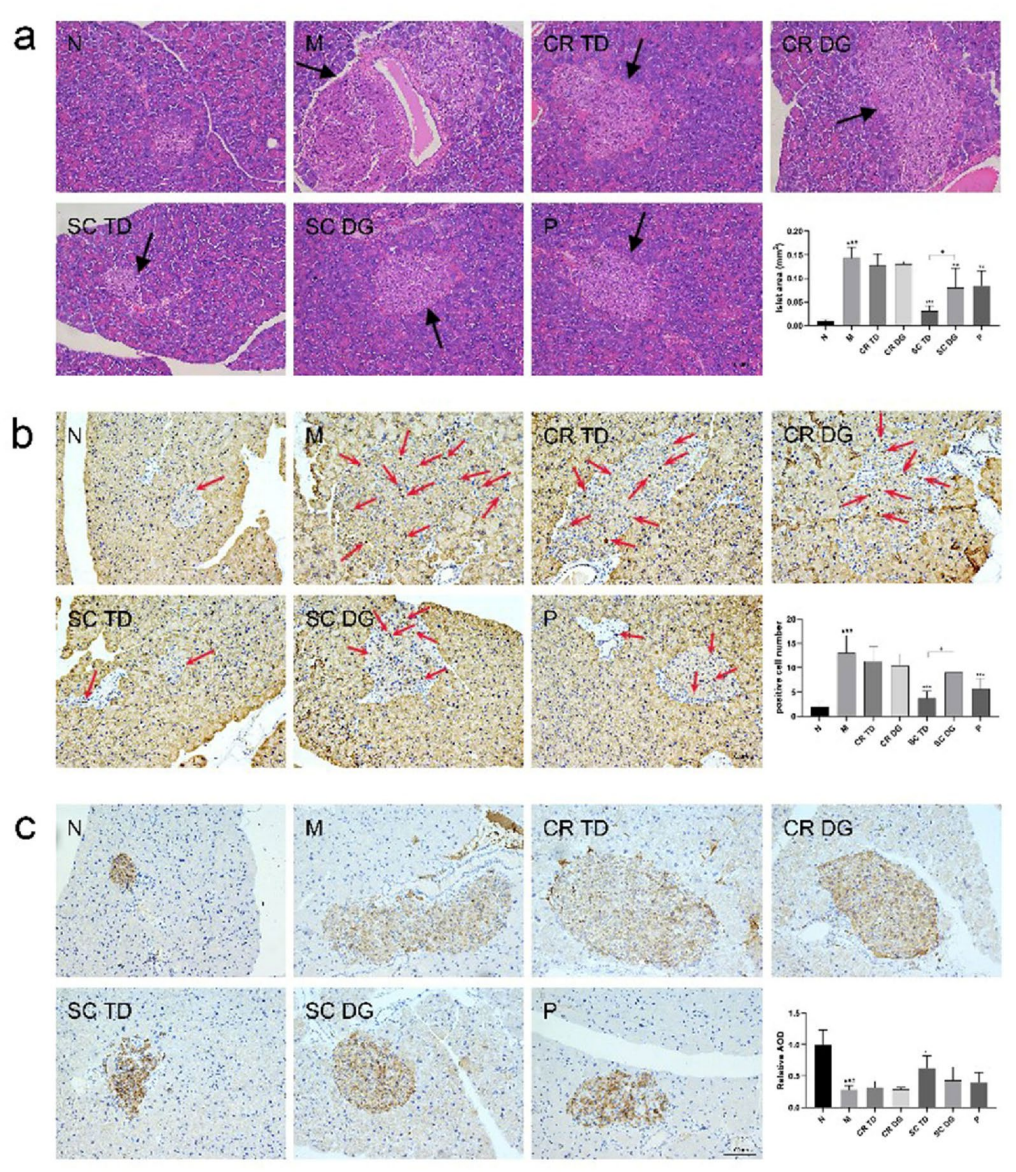
Effects of samples on morphology, apoptosis, and insulin secretion of pancreatic tissue in diabetic mice. **a** Morphology of pancreatic islets stained with hematoxylin and eosin (H&E), and the average islet size. Black arrows denote islets. **b** TUNEL staining and positive islet cell number. Red arrows denote apoptotic β-cells. **c** Representative images of insulin immunohistochemical staining and relative average optical density (AOD) values. Relative AOD means the average insulin of islet cells in the treated group relative to the N group. N: normal control group (*db/m* mice); M: model group (*db/db* mice); CR TD, CR DG, SC TD, SC DG, and P: the *db/db* mice treated with CR TD, CR DG, SC TD, SC DG, and metformin. Data were expressed as the mean ± SD (*n* = 6 mice per group). ^###^p < 0.001 versus N group; *p < 0.05, **p < 0.01, ***p < 0.001 versus M group; ^+^p < 0.05 versus SC TD group. Data were analyzed by one-way-ANOVA.

The hematoxylin and eosin (H&E) staining clearly revealed the compensatory hyperplasia of pancreatic islets in diabetic mice with obvious hypertrophy, malformation, and amyloid deposition, due to the inability of the pancreatic β-cells to compensate for the metabolic load of glucose. The Hematoxylin and Eosin (H&E) staining clearly revealed pronounced illustration of compensatory hyperplasia in pancreatic islets from diabetic mice, which was characteristically associated with significant hypertrophy, morphological anomalies, and an increase in amyloid deposition, caused by the inability of the pancreatic β-cells to compensate for the metabolic load of glucose. After 6 weeks of treatment, we found that the mice in the M group showed significant histopathological changes (Fig. 4a), such as the irregular arrangement of islet cells, the generation of vacuoles in the islets, blurred pancreatic acini boundaries, and increased size of islet cells (p < 0.001). The TUNEL staining assay revealed the significantly severe apoptosis of islet cells in *db/db* mice (p < 0.001) (Fig. 4b). The immunohistochemical insulin staining exhibited intense insulin staining in the N group and weak staining in the M group, and the average insulin per unit area in the islets of M group was lower than that of N group (p < 0.001) (Fig. 4c), suggesting that islets cells were markedly damaged in *db/db* mice. Compared with the M group, SC TD displayed the strongest amelioration of pathological changes, significantly reduced the size of islet cells (p < 0.001), decreased the apoptosis of islet cells (p < 0.001), and enhanced insulin secretion (p < 0.05). SC DG group showed almost the same islet cells size as the P group, but the effect on islet cells size and apoptosis of islet cells were markedly inferior to those of the SC TD group (p < 0.05). Although the CR TD and CR DG appeared to influence damaged islets as well, no significant variation in statistical values was observed. The above results showed that SC TD can significantly improve pancreatic injury in diabetic mice.

### Effects of samples on serum inflammatory factors

**Fig. 5:**
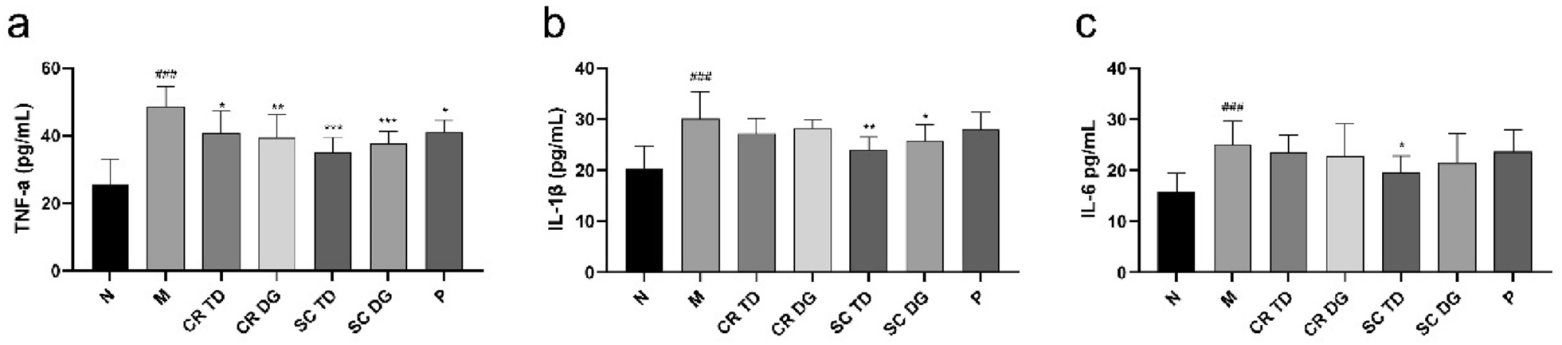
Effects of samples on the secretion of serum inflammatory factors in diabetic mice. **a** The levels of tumor necrosis factor-α (TNF-α), **b** interleukin-1β (IL-1β), and **c** interleukin-6 (IL-6) in serum of diabetic mice. N: normal control group (*db/m* mice); M: model group (*db/db* mice); CR TD, CR DG, SC TD, SC DG, and P: the *db/db* mice treated with CR TD, CR DG, SC TD, SC DG, and metformin. Data were expressed as the mean ± SD (*n* = 6 mice per group). ^###^p < 0.001 versus N group; *p < 0.05, **p < 0.01, ***p < 0.001 versus M group. Data were analyzed by one-way-ANOVA.

To determine the effect of all tested samples on inflammation, we measured the levels of serum tumor necrosis factor-α (TNF-α), interleukin-1β (IL-1β), and interleukin-6 (IL-6) in mice. The M group showed significantly higher TNF-α (Fig. 5a), IL-1β (Fig. 5b), and IL-6 (Fig. 5c) levels than the N group (p < 0.001). The influence of CR TD or CR DG on the above three inflammatory cytokines was basically the same, only showing a significant reduction in TNF-α (p < 0.05 and p < 0.01). Compared with the M group, the SC TD significantly decreased the TNF-α, IL-1β, and IL-6 levels in *db/db* mice (p < 0.05, p < 0.01, and p < 0.001), while SC DG reduced the levels of TNF-α and IL-1β (p < 0.05 and p < 0.001). These results indicated that compared with the SC DG, CR TD and CR DG, the SC TD had a more significant effect on the reduction in serum inflammatory factors in *db/db* mice.

### The samples inhibited oxidative stress in diabetic mice

**Fig. 6:**
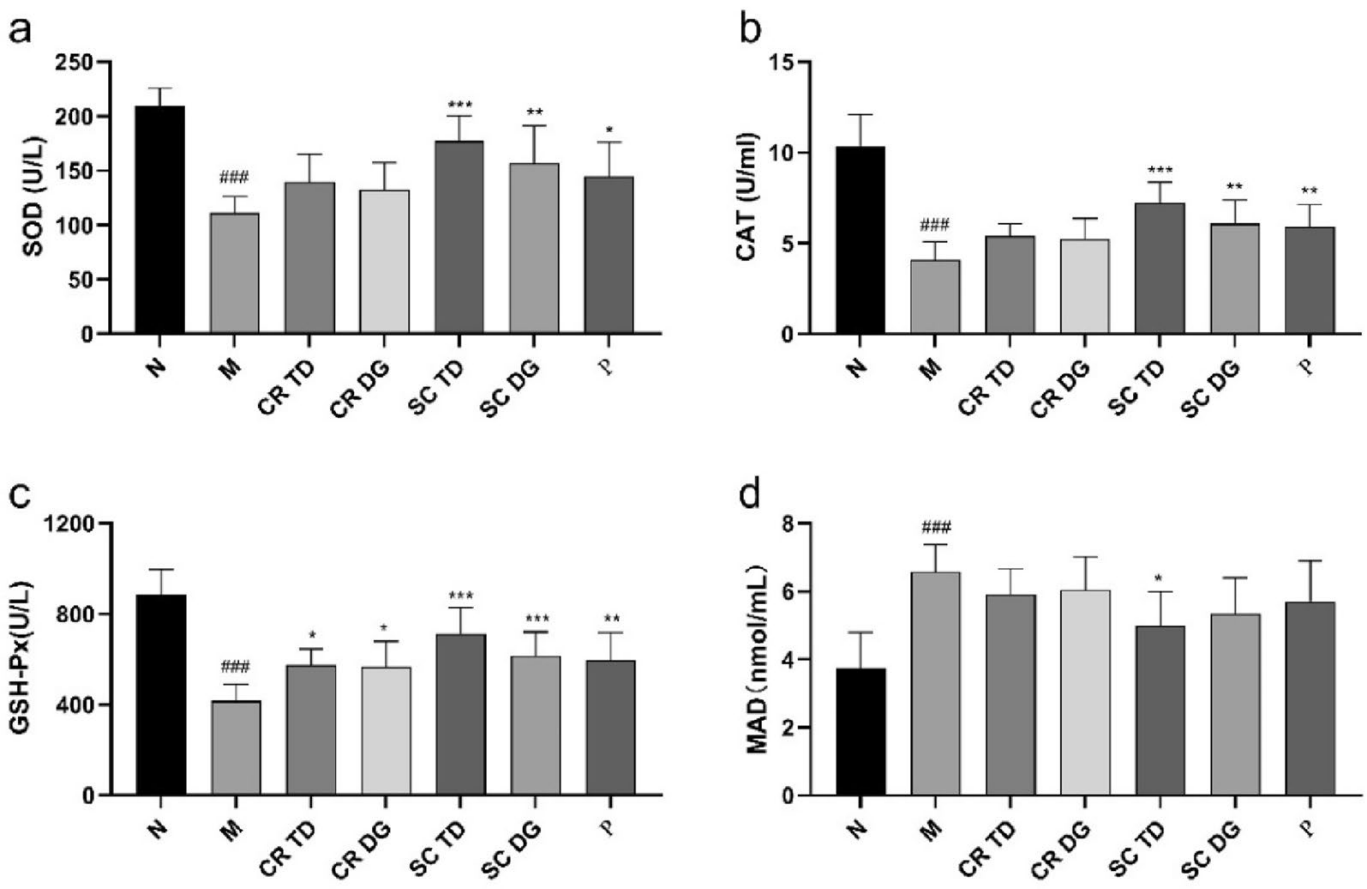
Reversing oxidative stress by samples in *db/db* diabetic mice. **a** The activities of superoxide dismutase (SOD), **b** catalase (CAT), **c** glutathione peroxidase (GSH-Px), and **d** the content of malondialdehyde (MDA) in the serum of diabetic mice. N: normal control group (*db/m* mice); M: model group (*db/db* mice); CR TD, CR DG, SC TD, SC DG, and P: the *db/db* mice treated with CR TD, CR DG, SC TD, SC DG, and metformin. Data were expressed as the mean ± SD (*n* = 6 mice per group6). ^###^p < 0.001 versus N group; *p < 0.05, **p < 0.01, ***p < 0.001 versus M group. Data were analyzed by one-way-ANOVA.

The antioxidant enzymes superoxide dismutase (SOD), catalase (CAT), and glutathione peroxidase (GSH-Px) which represent an anti-oxidative function in serum were assessed, as well as the content of oxidative product malondialdehyde (MDA). As shown in Fig. 6a–c, the activities of CAT, GSH-Px, and SOD were significantly lower in M group than in N group (p < 0.001), and the MDA (Fig. 6d) content in M group was much higher than in N group (p < 0.001), indicating that the antioxidative function of the *db/db* mice was impaired. However, the activity levels of CAT, GSH-Px, and SOD were increased to varying degrees in the serum of *db/db* mice after treatment compared with those in M group, a significant increase was observed in SC DG and P groups (P < 0.05 or P < 0.01), especially in SC TD group (P < 0.001), while a significant decrease in MDA content was observed only in SC TD group (P < 0.05).

### SC improved insulin resistance by activating Akt/AMPK/GLUT4 signaling pathways

**Fig. 7:**
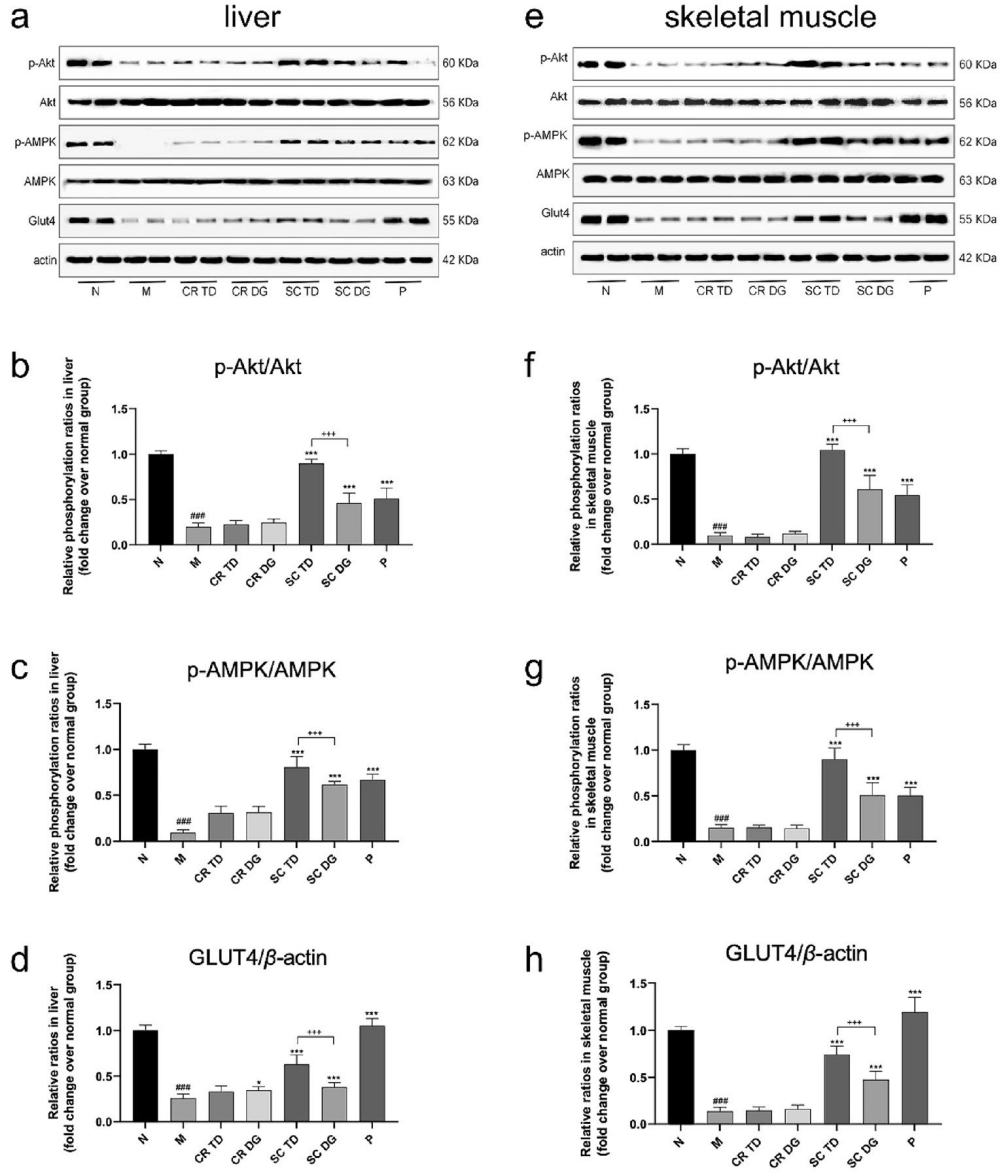
Western blotting analysis of protein expression of p-Akt, p-AMPK, and GLUT4 in liver and skeletal muscle tissues. The expression level of p-Akt, p-AMPK, and GLUT4 in liver (**a**) and skeletal muscle (**e**), quantitative analyses of p-Akt/Akt (b), p-AMPK/AMPK (**c**) and GLUT4/*β*-actin (**d**) in liver, quantitative analyses of p-Akt/Akt (**f**), p-AMPK/AMPK (**g**) and GLUT4/*β*-actin (**h**) in skeletal muscle  . N: normal control group (*db/m* mice); M: model group (*db/db* mice); CR TD, CR DG, SC TD, SC DG, and P: the *db/db* mice treated with CR TD, CR DG, SC TD, SC DG, and metformin. Data were expressed as the mean ± SD (*n* = 6 mice per group). ^###^p < 0.001 versus N group; *p < 0.05, **p < 0.01, ***p < 0.001 versus M group; ^+++^p < 0.001 versus SC TD group. Data were analyzed by one-way-ANOVA.

Insulin resistance is one of the defining characteristics of T2DM, primarily observed in the liver and skeletal muscle tissue [13]. To evaluate the effects of our samples on insulin resistance, the key signaling molecules related to insulin resistance, such as AKT, AMPK, and GLUT4 were preferentially investigated by western blotting (Fig. 7). We found that the protein expression of p-Akt, p-AMPK, and GLUT4 is significantly downregulated in M group compared with N group (p < 0.001). Compared with M group, above-mentioned proteins were prominently upregulated by SC and metformin treatment (p < 0.001). However, CR treatment only increased the expression of GLUT4 in liver tissue (p < 0.05), and not the expression of the other proteins. Furthermore, the results showed the upregulation of p-Akt, p-AMPK and GLUT4 expression by SC TD was more obvious than that by SC DG (p < 0.001), which echoes the above-mentioned observations. Based on the above evidence, the SC exhibited anti-T2DM effects mainly by activating AKT/AMPK/GLUT4 signaling pathways to improve insulin resistance.

## Discussion

In the present study, a systematic comparative study on the quality markers and hypoglycemic effects of CR or Scutellaria–coptis (SC) herb pair in the forms of TD and DG was carried out by UPLC-PDA method and T2DM mouse model. This study indicated that the contents of the main alkaloids in CR DG samples were significantly lower than those in CR TD samples, which were prepared by the same batches of raw materials. For SC samples, the contents of ten major compounds were reduced significantly relative to the single herb in the TD sample but not in the DG sample, resulting in the relative ratio of alkaloids to flavonoids being determined as 1:1 in SC TD and 1:2 in SC DG. The animal experiments showed that the CR DG had almost the same hypoglycemic effect as CR TD when administered alone at a dose equivalent to 3 g decoction pieces/kg for 6 weeks. However, compared with SC DG (equivalent to 6 g decoction pieces/kg), the present study indicated that SC TD (equivalent to 6 g decoction pieces/kg) had a stronger anti-T2DM effect.

Recently, some studies have begun to evaluate the chemical similarity and bioequivalence of DG and TD to address their substitutability. Liang and Zhang respectively conducted the consistency evaluation of SR DG and CR DG with corresponding TD in chemistry and activity *in vitro* [14, 15]. The DG samples used in the above two studies were produced by different pharmaceutical companies, while the TD samples were prepared using the raw materials collected from the market. Thus, the different quality of the original materials might be one of the reasons for the unsatisfactory comparison results. To avoid this limit, we collected several batches of decoction pieces which were used to produce DG samples from pharmaceutical companies, and conducted this comparative study of DG and TD samples that were homologous. For the single herbs CR or SR, TD samples showed generally higher contents of target compounds than DG samples. This difference might be caused by the distinctions between commercial scale and laboratory scale processing. Meanwhile, the variations in extraction procedures or parameters applied in a laboratory compared to that used in industrial production might be the reason for different extraction rates. The results also reminded us to pay attention to equivalent conversion when using DG instead of TD. For the SC herb couple, the complex precipitation during boiling lead to the significantly lower contents of ten investigated components in SC TD than SR TD or CR TD, which was consistent with reported data [16, 17]. However, that remarkable reduction in contents has not appeared in the DG sample, indicating that the interaction between the crude drugs occurred during the boiling process rather than simply mixing and dissolving. Moreover, the relative ratio of main alkaloids to flavonoids varied from 1:1 in SC TD to 1:2 in SC DG. Due to the alkaloids and flavonoids being the main active compounds of the SC, the variation in the relative ratio of active constituents may lead to differences in activity.

Based on the clinical efficacy of CR and SC in treating diabetes mellitus, this study compared the therapeutic effects of their DG and TD samples in vivo for the first time. The *db/db* mice, commonly used in the study of T2DM [18], were chosen to evaluate the anti-diabetic effects of CR TD, CR DG, SC TD, and SC DG. Consistent with previous studies [8, 9, 11, 19], present study showed that CR and SC significantly decrease body weight and FBS levels, and improve insulin resistance in T2DM animal models. There were no statistically significant variations observed in body weight, FBS, OGTT, ITT, FINS, and HOMA-IR between CR TD and CR DG groups, suggesting that CR TD and CR DG (equivalent to 3 g decoction pieces/kg) had almost the same hypoglycemic effect in *db/db* mice. However, the clinical application of CR is mainly in the form of multi-herbal formula. It is noteworthy that the enhancing effect of CR and SR was observed not only in TD samples, but also in DG samples. However, SC TD (equivalent to 6 g decoction pieces/kg) had a stronger anti-T2DM effect than SC DG. Although the reduction in body weight and FBS was similar between the two groups, the mice treated with SC TD had lower HOMA-IR and showed stronger improvement effects of glucose tolerance and insulin tolerance. According to the compatibility theory of traditional Chinese medicine, different compatibility proportions of crude drugs or active ingredients can affect their efficacy and toxicity [20–22]. Thus, the variation in the antidiabetic effect of SC TD and SC DG might be attributed to the changed compatibility proportions of active ingredients. Zhang conducted an investigation to examine the impact of the baicalin-berberine combination on glucose uptake *in vitro*. The findings revealed an additive interaction between baicalin and berberine at lower baicalin doses, while higher doses of baicalin exhibited an antagonistic effect [23]. The present study showed that baicalin content in SC DG was more than 2-fold than that in SC TD. The high contents of baicalin might have a negative influence on the hypoglycemic effects of berberine. Besides, studies have indicated that baicalein could ameliorate Type 2 Diabetes through the inhibition of the MAPKs pathway and the activation of the IRS1/PI3K/Akt pathway. Therefore, we speculated that the observably higher content of baicalein in SC TD potentially serve as another determinant for the more pronounced hypoglycemic effects of SC TD compared to SC DG [24]. However, the detailed reasons for the variation in the antidiabetic effect of SC TD and SC DG could be complex and remain to be further explored.

The pancreatic islets, responsible for producing endocrine hormones that regulate metabolic homeostasis, play a crucial role in the pathogenesis of diabetes. Research has demonstrated that pancreatic β-cells possess the ability to adapt and meet heightened metabolic demands and insulin requirements, thus maintaining optimal blood glucose levels [25–27]. However, in the case of T2DM, pancreatic β-cells become unable to secrete sufficient insulin to adequately address the metabolic needs of insulin-responsive tissues [28–30]. This study showed that CR TD and CR DG slightly improved pancreatic injury in diabetic mice. SC DG significantly inhibited the apoptosis of pancreatic β-cells, and improved the compensatory proliferation of pancreas, but it had no obvious effect on the secretory function of pancreas. Consistent with the effect of relieving T2DM, SC TD showed significant improvement in promoting the normalization of pancreatic structure and secretion function. Furthermore, the involvement of inflammation, oxidative stress, endoplasmic reticulum stress in beta cell dysfunction, and apoptosis has been documented [31]. Several studies have suggested that the bioactivities of CR or SR including antioxidant and anti-inflammatory functions may facilitate its antidiabetic functions [2]. Our research showed that CR and SC in two dosage forms exhibited different degrees of improvement in inflammatory reactions and oxidative stress in *db/db* mice. Similarly, SC TD had the strongest effect in regulating the secretion of related inflammatory factors and the activity of antioxidant enzymes. All the above results showed that the superior anti-T2DM effect of SC TD is partly due to its inhibition of inflammation and oxidative stress, and improvement of pancreatic structure and secretion function.

Insulin resistance which commonly occurrs in muscle and liver is another major pathological factor of T2DM. GLUT4, a major glucose transporter, mediates glucose uptake and plays a key role in the maintenance of whole-body glucose homeostasis. Its defective expression or translocation leads to the decreased insulin-stimulated glycogen synthesis, thereby contributing to insulin resistance in individuals with type 2 diabetes. The continuous transportation of glucose by GLUT4 is primarily regulated through insulin-dependent signaling pathways, such as PI3K/Akt, as well as insulin-independent pathways involving AMPK [32]. Abnormal expression of Akt and AMPK influence the expression level of GLUT4, and further lead to T2DM. Therefore, Akt/AMPK/GLUT4 signaling pathway may be potentially therapeutic target for T2DM. Findings from previous researches have shown that the CR, SR and SC ameliorated T2DM via up-regluating the expression of p-Akt and key targets of MAPK pathway [8]. Herein, to comfirm whether CR and SC improved T2DM by activating the Akt/AMPK/GLUT4 signaling pathway directly, we detected the protein expression of p-Akt, p-AMPK and GLUT4 in the liver and muscle. The results showed that CR TD had no significant effect on the expression of p-Akt, p-AMPK, and GLUT4 protein, while CR DG increased the expression of GLUT4 in liver tissue of diabetic mice, indicating that the hypoglycemic effect of CR may not depend on the activation of classical Akt signaling pathway. Unlike CR, SC significantly increased the expression levels of p-Akt, p-AMPK, and GLUT4 protein in liver and skeletal muscle tissue, which suggests that SC may promote GLUT4 translocation and increase glucose uptake by activating insulin signaling and AMPK signaling. Previous research has highlighted baicalin, a chief compound in SR, as a pivotal agent in alleviating insulin resistance by enhancing the expression of p-MAPK, p-Akt, and GLUT4 [33]. Therefore, we speculated that SC stimulates Akt/AMPK/GLUT4 pathway through the influence of SR rather than CR. Another probable factor to consider is the potential synergistic effect of CR and SR. This is supported by the observation that the SC extracts displayed a more notable impact on the MAPK and Akt pathways in T2DM rats compared to CR and SR, as documented in existing literature [8]. Further investigation is necessary to elucidate the precise factors contributing to the disparity in the activation of Akt/AMPK/GLUT4 pathways between CR and SC. Additionally, the expression levels of p-Akt, p-AMPK and GLUT4 protein in the SC TD treatment group were significantly higher than those in the SC DG group, aligning with the observed reduction in fasting blood glucose levels and enhancement of insulin resistance.

## Conclusions

The present results showed the content difference in marker compounds between dispensing granules (DG) and traditional decoction (TD) of Coptidis Rhizoma (CR), as well as Scutellaria–coptis herb couple (SC). The animal experiments showed that the CR DG had almost the same hypoglycemic effect as CR TD when administered alone at a dose equivalent to 3 g decoction pieces/kg for 6 weeks. Compared with SC DG (equivalent to 6 g decoction pieces/kg), SC TD showed a better trend in terms of ameliorating T2DM via inhibiting inflammation and oxidative stress, ameliorating pancreatic structure and secretion function, and activating Akt/AMPK/GLUT4 signaling pathways. This preliminary study revealed that the interaction caused by the different preparation processes of SC DG and SC TD lead to the differences in the chemical contents and hypoglycemic effects. However, whether the above differences in pharmacology may lead to significant differences in clinical practice requires further investigation. Since DG has been widely used in clinical practice, we hope that the variations in the chemical composition and efficacy between DG and TD could also be noticed for other herbal medicines.

### Supplementary Information

**Additional file 1: Table S1.** Detailed information of dispensing granules and corresponding decoction pieces; **Table S2.** Extraction rates of CR and SR samples (%); **Table S3.** Contents of main alkaloids in CR samples (mg/g decoction pieces); **Table S4.** Contents of main flavonoids in SR samples (mg/g decoction pieces).

## Data Availability

The datasets supporting the conclusions of this article are included within the article and its additional files.

## Abbreviations

DG: Dispensing granules
TD: Traditional decoction
CR: Coptidis Rhizoma
SR: Scutellariae Radix
SC: Scutellaria–coptis herb couple
T2DM: Ttype 2 diabetes mellitus
TCMs: Traditional Chinese medicines
UPLC-PAD: Ultra-performance liquid chromatography coupled with a photodiode array detector
FBG: Fasting blood glucose
HOMA-IR: Homeostasis model assessment insulin resistance index
OGTT: Oral glucose tolerance tests
ITT: Iinsulin tolerance tests
ELISA: Enzyme-linked immunosorbent assay
WB: Western blot analysis
