# Supplementary material for: Comparative study on main compounds and hypoglycemic effects of dispensing granules of Coptidis Rhizoma and Scutellaria–Coptis herb couple with traditional decoction
Source: Chin Med. 2023 Oct 31;18:141. doi: 10.1186/s13020-023-00848-z (PMC10617169; doi:10.1186/s13020-023-00848-z)
Supplement: Supplementary file 1 — Additional file 1: Table S1. Detailed information of dispensing granules and corresponding decoction pieces; Table S2. Extraction rates of CR and SR samples (%); Table S3. Contents of main alkaloids in CR samples (mg/g decoction pieces); Table S4. Contents of main flavonoids in SR samples (mg/g decoction pieces). [file 13020_2023_848_MOESM1_ESM.docx]

**Additional Material**

**Comparative study on main compounds and hypoglycemic effects of** **dispensing granules of Coptidis Rhizoma and** **Scutellaria–Coptis herb couple with traditional decoction**

Huan-Huan Yu^1,2^, Hui-Lan Tang^2^, Guang Hu^3^, Zhu Chen^2^, Mu-Dan Guo^2^, Bo Jiang^2^, En Zhang^2*^, Chang-Hua Hu^1*^

^1^ College of Pharmaceutical Sciences, Southwest University, Chongqing 400715, China

^2^ NMPA Key Laboratory for Quality Monitoring of Narcotic Drugs and Psychotropic Substances. Chongqing Institute for Food and Drug Control, Chongqing 401121, China

^3^ School of Pharmacy and Bioengineering, Chongqing University of Technology, Chongqing 400054, China;

***** Corresponding author

zhangen@cqifdc.org.cn (En Zhang);

chhhu@swu.edu.cn (Changhua Hu);

1. **The detailed information of samples.**

The 8 batches of Coptidis Rhizoma dispensing granules and 7 batches of Scutellariae Radix dispensing granules were collected from different pharmaceutical manufacturers in China, while the decoction pieces used to prepare CR DG01-06 and SR DG01-05 were respectively obtained to prepare TD samples. The detailed information was shown in Table S1.

**Table S1.** Detailed information of dispensing granules and corresponding decoction pieces.

| Batch number of DG | Corresponding decoction piece | Manufacturer | Each gram of dispensing granules equivalent to the amount of medicinal materials (g/g) |
| --- | --- | --- | --- |
| CR DG01 (CAHL0101) | CR 01 | A | 4.5 |
| CR DG02 (CAHL0102) | CR 02 | A | 4.5 |
| CR DG03 (CAHL0103) | CR 03 | A | 4.5 |
| CR DG04 (CAHL0201) | CR 04 | B | 4.5 |
| CR DG05 (CAHL0202) | CR 05 | B | 4.5 |
| CR DG06 (CAHL0203) | CR 06 | B | 4.5 |
| CR DG07 (CAHL0301) | - | B | 4.5 |
| CR DG08 (CAHL0302) | - | B | 4.5 |
| SR DG01 (CAHQ0101) | SR 01 | A | 2.2 |
| SR DG02 (CAHQ0102) | SR 02 | A | 2.2 |
| SR DG03 (CAHQ0201) | SR 03 | B | 2.2 |
| SR DG04 (CAHQ0202) | SR 04 | B | 2.2 |
| SR DG05 (CAHQ0203) | SR 05 | B | 2.2 |
| SR DG06 (CAHQ0301) | - | B | 2.2 |
| SR DG07 (CAHQ0302) | - | B | 2.2 |

-: The same batches of decoction pieces have not been collected.

1. **Extraction ratios of traditional decoction**

The crude Coptidis Rhizoma (CR01-06) and Scutellariae Radix (SR01-05) were crushed and made into traditional decoction (TD) according to the Standard for Management of TCM Decocting Room in Medical Institutions. The extraction ratios of CR and SR samples were shown in Table S2.

**Table S2.** Extraction rates of CR and SR samples (%)

| Drugs | 01 | 02 | 03 | 04 | 05 | 06 |
| --- | --- | --- | --- | --- | --- | --- |
| CR | 26.47 | 28.83 | 28.33 | 30.21 | 33.70 | 33.24 |
| SR | 53.79 | 57.47 | 58.25 | 58.37 | 51.52 | - |

-: The decoction pieces have not been collected.
